# Missing Cells: Pathophysiology, Diagnosis, and Management of (Pan)Cytopenia in Childhood

**DOI:** 10.3389/fped.2015.00064

**Published:** 2015-07-13

**Authors:** Miriam Erlacher, Brigitte Strahm

**Affiliations:** ^1^Division of Pediatric Hematology and Oncology, Department of Pediatrics and Adolescent Medicine, University Medical Center of Freiburg, Freiburg, Germany; ^2^Freiburg Institute for Advanced Studies, University of Freiburg, Freiburg, Germany

**Keywords:** cytopenia, childhood, bone marrow failure, hypocellular bone marrow, myelodysplastic syndrome, refractory cytopenia of childhood, severe aplastic anemia

## Abstract

Peripheral blood cytopenia in children can be due to a variety of acquired or inherited diseases. Genetic disorders affecting a single hematopoietic lineage are frequently characterized by typical bone marrow findings, such as lack of progenitors or maturation arrest in congenital neutropenia or a lack of megakaryocytes in congenital amegakaryocytic thrombocytopenia, whereas antibody-mediated diseases such as autoimmune neutropenia are associated with a rather unremarkable bone marrow morphology. By contrast, pancytopenia is frequently associated with a hypocellular bone marrow, and the differential diagnosis includes acquired aplastic anemia, myelodysplastic syndrome, inherited bone marrow failure syndromes such as Fanconi anemia and dyskeratosis congenita, and a variety of immunological disorders including hemophagocytic lymphohistiocytosis. Thorough bone marrow analysis is of special importance for the diagnostic work-up of most patients. Cellularity, cellular composition, and dysplastic signs are the cornerstones of the differential diagnosis. Pancytopenia in the presence of a normo- or hypercellular marrow with dysplastic changes may indicate myelodysplastic syndrome. More challenging for the hematologist is the evaluation of the hypocellular bone marrow. Although aplastic anemia and hypocellular refractory cytopenia of childhood (RCC) can reliably be differentiated on a morphological level, the overlapping pathophysiology remains a significant challenge for the choice of the therapeutic strategy. Furthermore, inherited bone marrow failure syndromes are usually associated with the morphological picture of RCC, and the recognition of these entities is essential as they often present a multisystem disease requiring different diagnostic and therapeutic approaches. This paper gives an overview over the different disease entities presenting with (pan)cytopenia, their pathophysiology, characteristic bone marrow findings, and therapeutic approaches.

## Introduction

(Pan)cytopenia in childhood can be caused by a variety of underlying diseases, including hematological and non-hematological entities. Overlapping phenotypes and pathophysiologies pose a major diagnostic challenge. However, an accurate and rapid diagnosis is essential for adequate therapy planning, surveillance, and genetic counseling. Bone marrow (BM) analysis is of special importance for the diagnostic work-up of cytopenias affecting one or more lineages. In the following, we will describe the individual disorders, their underlying pathophysiology and clinical characteristics, as well as typical BM findings.

## The Hierarchy of Hematopoiesis

**Figure 1:**
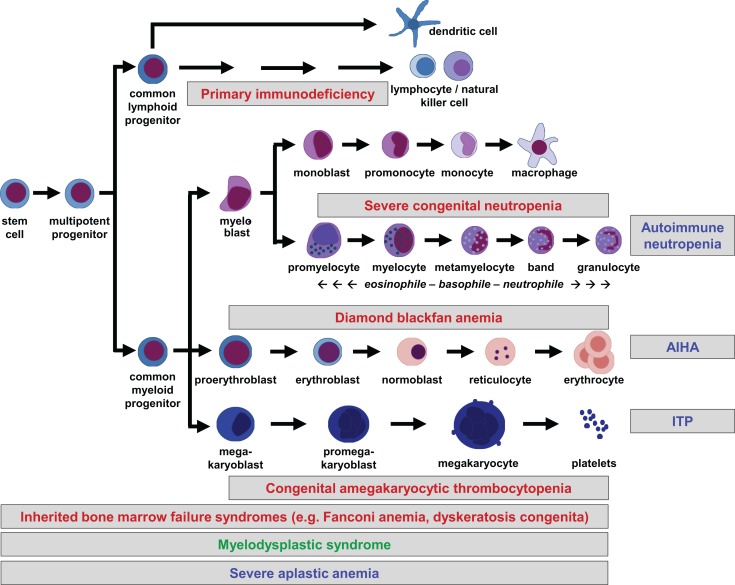
**Hierarchical tree of human hematopoiesis**. Disturbances leading to cytopenias can affect single or multiple lineages and be caused by cell-intrinsic or extrinsic mechanisms. Intrinsic defects are caused by inherited or acquired mutations, while extrinsic defects can be caused by autoreactive lymphocytes. A selection of frequent pediatric disorders is shown. Labeling: inherited defects: red; acquired mutations: green; autoimmune disorders: blue. AIHA, autoimmune hemolytic anemia; ITP, idiopathic thrombocytopenic purpura.

All peripheral blood cells originate from hematopoietic stem cells (HSCs) that reside in fetal liver and fetal or adult BM. HSCs are able to self-renew, which is essential for maintenance of lifelong hematopoiesis. They do not commit directly to single lineages, but instead differentiate into multipotent progenitors, common lymphoid and (erythro-) myeloid progenitors, which in turn give rise to more differentiated precursor cells. Lineage commitment is determined by a delicate network of transcription factors and epigenetic mechanisms that establish differentiation into the corresponding lineage while suppressing maturation toward other lineages (1). The hierarchical tree of hematopoiesis is depicted in Figure 1, but there is evidence that such a linear and rigid model is oversimplified (2). Also, a specific HSC subset has been reported to directly evolve into megakaryocytes, at least in the murine system (3). Depending on the differentiation stage affected, disturbances can occur in single or multiple lineages (Figure 1).

## Signs and Symptoms of Cytopenia

The condition caused by low erythrocyte numbers and hemoglobin concentration is called anemia and is clinically characterized by paleness, weakness, and general malaise. Severe anemia due to rapid hemoglobin drop (i.e., blood loss, hemolysis) may lead to cardiovascular symptoms such as tachycardia and arterial hypotension. Anemia due to bone marrow failure usually has insidious manifestation. Reticulocytosis points toward active red cell production while lack of reticulocytes is indicative for insufficient erythropoiesis. Other informative parameters are mean corpuscular volume (MCV) and HbF levels as signs of stress hematopoiesis or low haptoglobin with increased unconjugated bilirubin in the presence or absence of a positive antiglobulin test indicating hemolysis (4).

Platelets are fragments of membrane and cytoplasm derived from megakaryocytes and important for hemostasis. Thrombocytopenia is characterized by cutaneous and mucosal bleeding signs, such as epistaxis, petechia, and purpura. Platelet size and morphology can help classifying the underlying disease. Platelets are abnormally small in patients with Wiskott–Aldrich syndrome (WAS) and large in patients with Bernard–Soulier syndrome or May–Hegglin anomaly (5). Platelet size diminishes over time, and therefore higher platelet volume may indicate the presence of less mature platelets in cases of increased degradation (e.g., idiopathic thrombocytopenic purpura, ITP) (6).

Lack of neutrophils, neutropenia, is characterized by an increased susceptibility to bacterial infections. Risk of infections correlates with granulocyte counts, with severe neutropenia (<500/μl) being associated with a high risk of life-threatening infections (7). Depending on the extent of neutropenia and the frequency of severe infections, prophylactic antibiotic treatment and/or G-CSF application might be considered. Response to G-CSF is indicative for the myelopoietic capacity of the BM.

In line with the many different types and developmental stages of lymphocytes, lymphopenia can affect an entire lineage (i.e., B, T, and/or NK cells) or individual mature cell types (e.g., regulatory T cells). Type of lacking cells as well as abundance of other lymphocyte types determine the phenotype of the individual disease. Phenotypes can be highly variable and range from (severe) combined immunodeficiency to mildly increased susceptibility to specific infections. Primary immune diseases can also be associated with immune dysregulation (8).

Neonatal cytopenias are particularly challenging due to the extremely diverse spectrum of differential diagnoses. Impairment of fetal erythropoiesis or thrombopoiesis due to inherited defects or prenatal infections can result in hydrops fetalis or perinatal hemorrhagic complications, respectively. Ineffective fetal hematopoiesis within the BM can lead to persistent extramedullary hematopoiesis in liver, spleen, and occasionally in the skin (9). Affected newborns are characterized by hepatosplenomegaly and may have skin infiltrations (so called “blueberry muffin” babies). The latter condition is indicative for hemolytic diseases, malignant BM infiltrations, infantile malignant osteopetrosis, or congenital infections (10). Neonatal erythroderma might indicate the presence of severe immune dysregulation as seen in Omenn’s syndrome, a hyperinflammatory variant of leaky SCID (11).

A detailed medical history is essential in all cases of cytopenias and should include details of concomitant health problems, susceptibility to bleeding or infections, previous exposure to drugs or toxins, and recent foreign travel. Family history should focus on hematological and immune disorders, cancer susceptibility, or unexplained infant deaths. Thorough physical examination should include the assessment of signs of lymphoproliferation (i.e., hepatosplenomegaly and lymphadenopathy), as well as dysmorphic features, and stigmata, indicating the presence of a syndromic disease.

## Cytopenia Affecting a Single Lineage

In principle, single lineage-cytopenias may be caused by insufficient production or premature depletion of mature cells of the respective hematopoietic lineage. In the majority of cases, the latter is mediated by autoreactive antibodies, resulting in ITP, autoimmune neutropenia (AIN), or autoimmune hemolytic anemia (AIHA), respectively (12). Frequently, autoantibodies are formed as a reaction to infections or vaccinations due to the so-called “molecular mimicry” between pathogen components and blood cell antigens (13). Similarly, heparin-induced thrombocytopenia (HIT) is caused by antibodies specific for heparin-binding platelets (14). Generally, these disorders show normal BM morphology or hyperactive hematopoiesis in the corresponding lineage. Most tests for autoantibodies have a low sensitivity and specificity, and are therefore not required for the confirmation of the diagnosis, except for AIHA (15). Since the BM is normal in ITP or AIN and thus has the capacity of increased cell production if stressed, signs and symptoms are frequently milder compared to patients with hypoproliferative deficiencies of these cell lineages. Even in the complete absence of peripheral granulocytes, patients rarely suffer from life-threatening infections. In contrast to these clinically mostly benign autoimmune disorders, AIHA, especially in childhood, can be life-threatening because of rapid hemoglobin decline (16).

Furthermore, a premature destruction of red blood cells may be caused by defective hemoglobin, metabolic enzymes, or membrane components. These syndromes can be diagnosed by hemoglobin electrophoresis, determination of enzymatic activity or osmotic fragility tests, respectively, and will not be discussed here in more detail. Increased destruction of platelets and the subsequent risk of bleeding are also characteristic features of inherited disorders, such as Wiskott–Aldrich syndrome (WAS) and X-linked thrombocytopenia (XLT). While platelet generation has been shown to be unaffected in this syndrome, cytoskeletal changes occurring in circulating platelets lead to their premature degradation (17).

Similar to the single lineage-cytopenias caused by premature cell loss, disorders with inadequate cell generation can affect all individual lineages, i.e., megakaryopoiesis, erythropoiesis, and myelopoiesis. These disorders, their pathophysiologies, diagnostic characteristics, and symptoms are summarized in Table 1. Disorders with insufficient generation of lymphocytes result in (severe) combined or milder immunodeficiencies. Since they require distinct diagnostic approaches and therapies, these syndromes are beyond the scope of this review and were discussed elsewhere (18).

**Table 1:** **Syndromes characterized by inadequate formation of mature blood cells resulting in single lineage cytopenia**.

Disease/syndrome	Mutated gene	Pathogenesis	Extrahematological features	Hematopoietic phenotype	Bone marrow morphology	Therapeutic options	Reference

**Severe congenital neutropenia (SCN)**	*ELANE*, *HAX1*, *GFI1, G6PC3, p14*, and others	Differentiation block, myelocyte apoptosis	Depending on disease (G6PC3: heart/uro-genital/facial anomalies)	Severe neutropenia, risk of leukemia	Maturation arrest at promyelocyte stage	G-CSF, antibiotic prophylaxis, HSCT, leukemia surveillance	(19–21)

**Cyclic neutropenia**	*ELANE*	Cyclic increase in myelocyte apoptosis	None	Cyclic neutropenia, normal blood in intervals	Intermittendly similarities to SCN	G-CSF	(22)
**Glycogen storage disease type 1b**	Glucose-6-phosphate translocase	Impaired glucose formation from glycogen	Hypoglycemia, increased hepatic and renal glycogen storage	Neutropenia, granulocyte dysfunction	Normal	G-CSF	(23)

**Shwachman Diamond syndrome (SDS)**	*SBDS*	Ribosome assembly and function and many other cellular functions	Exocrine pancreatic insufficiency, skeletal abnormalities, growth retardation	Neutropenia, pancytopenia (25%), risk of leukemia	Maturation arrest of myelopoiesis, hypocellular bone marrow	G-CSF, HSCT	(24)

**Congenital dyserythropoietic anemia (CDA)**	*CDAN1*, *SEC23B*, *KIF23*, *KLF1*, and others	Ineffective erythropoiesis	Iron overload	Anemia	Dyserythropoetic maturation of erythroblast	RBC transfusions, chelation therapy	(25)

**Diamond-Blackfan anemia (DBA)**	Ribosomal genes (*RPS19, RPL5, RPL11, RPL35A*, and others)	Ribosomopathy	Thumb malformations and craniofacial abnormalities	Macrocytic anemia, variable neutrophils, and platelet numbers, risk of leukemia	Paucity of erythroid precursors	Steroids, RBC transfusions, chelation therapy, HSCT	(26)

**Thalassaemia major**	Globin genes (*HBA1*, *HBA2*, *HBB*)	Unbalanced synthesis of globins	Bone deformities, iron overload	Anemia due to ineffective hematopoiesis and hemolysis, extramedullar hematopoiesis	Hyperplastic erythropoiesis, apoptosis of erythroid precursors	RBC transfusions, chelation therapy, HSCT	(27)

**Pure red cell aplasia (PRCA)**	Unknown	Unknown (autoimmune?)	None	Anemia	Absence of erythroblasts (maturation arrest)	Transfusions, immunosuppression	(28)

**Anemia of renal failure**	None	Insufficient erythropoietin production	Depending on underlying disease	Normocytic anemia	Uncharacteristic findings	Erythropoietin substitution	(29)

**Anemia of chronic inflammation**	None	Hyperinflammation, hepcidin deregulation	Depending on underlying disease	Normo- to microcytic anemia	Uncharacteristic findings	Treatment of underlying disease	(30)

**Congenital amegakaryocytic thrombocytopenia (CAMT)**	*MPL*	Megakaryopoiesis unresponsive to thrombopoietin	None	Thrombocytopenia, pancytopenia, risk of leukemia	Near-absence of megakaryocytes	Transfusions, HSCT	(31)

**Thrombocytopenia with absent radius (TAR)**	Chr. 1q21.1 deletion (*RBM8A?*)	Insufficient megakaryopoiesis (unknown cause)	Absence of radius (thumb present), cardiovascular and gastrointestinal malformations	Thrombocytopenia, risk of leukemia	Decreased or absent, small megakaryocytes with vacuolization	Transfusions, HSCT	(32)

**Familial platelet disorder (FPD)**	*Runx1*	Defective expression of RUNX1 targets (e.g., *MPL*)	None	Mild thrombocytopenia, high risk of MDS/leukemia	Normal or dysplastic signs	Transfusions, HSCT, leukemia surveillance	(33)

**Bernhard–Soulier syndrome**	*GP1BA*, *GP1BB*	Defective platelet formation, reduced platelet lifespan	None	Thrombocytopenia, increased platelet size	Increased megakaryopoiesis	Transfusions	(34)

**May–Hegglin anomaly**	*MYH9*	Defective megakaryocyte maturation	Sensineural deafness, cataract, nephritis	Mild thrombocytopenia, increased platelet size	Inclusion bodies in neutrophils	Symptomatic treatment	(5)

*ALL, acute lymphoblastic leukemia; AML, acute myeloid leukemia; G-CSF, granulocyte colony-stimulating factor; HSCT, hematopoietic stem cell transplantation; MDS, myelodysplastic syndrome; MPN, myeloproliferative neoplasia; RBC, red blood cells*.

## When More Lineages are Affected

Cytopenias affecting two or three blood lineages, the latter called pancytopenia (greek $\pi \overset{˜}{\alpha }\nu$, *pan* = involving all), can also be caused by deregulation of either cell generation or degradation. As an additional layer of classification, the disorders can be divided into inherited or acquired BM failures. It is important to emphasize that inherited BM failure syndromes (IBMFS) as well as acquired BM failure can present in all age groups, and that in some cases an isolated cytopenia preceeds the development of pancytopenia. This requires particular attention and repeated reevaluation by the attending physician or hematologist. Isolated thrombocytopenia can initially be diagnosed as ITP, but additional anemia or neutropenia over time might indicate the presence of systemic autoimmune disorders such as systemic lupus erythematosus (SLE). Similarly, isolated neutropenia can be considered to be secondary to an infection, but the subsequent decrease of other blood cells should rapidly lead to BM analysis. In the following, we will describe the different forms of pancytopenia in more detail.

## Pancytopenia as Consequence of Autoimmunity and Immune Dysregulation

While single-line immune-mediated degradation of platelets, erythrocytes, or neutrophils is frequent, combinations thereof are rare and often serious (35, 36). The combination of AIHA and thrombocytopenia, occurring either simultaneously or sequentially, is called Evans syndrome and is often a manifestation of the autoimmune lymphoproliferative syndrome (ALPS) (37). ALPS is caused by germline or somatic mutations in *FAS*, *FASL*, or *CASP10*, resulting in insufficient apoptosis of activated autoreactive lymphocytes via the extrinsic pathway (38, 39). Rarely, ALPS can be caused by *RAS* mutations resulting in defective intrinsic apoptosis of lymphocytes (40). Concomitant lymphoproliferation is characteristic for ALPS. Similarly, cytopenias can occur in acquired, multifactorial autoimmune syndromes such as SLE or the primary antiphospholipid syndrome (36). Germline syndromes characterized by autoimmunity are the IPEX (immunodysregulation polyendocrinopathy enteropathy X-linked) syndrome caused by lack of regulatory T cells and the APECED (autoimmune polyendocrinopathy-candidiasis-ectodermal dystrophy) syndrome caused by insufficient induction of central (thymic) tolerance (41, 42). Other primary immunodeficiencies frequently leading to autoimmune cytopenia are leaky SCID, WAS, hyper-IgM syndrome, and common variable immunodeficiency (CVID) (36). Indicative of the presence of such syndromes are lymphoproliferation, autoantibodies, oligoclonal T cells, increased complement consumption, and signs of autoimmunity or autoinflammation affecting other organs (i.e., dermatitis, glomerulonephritis, and inflammatory bowel disease). Bone marrow findings are usually not characteristic and merely indicate increased cellular turnover.

Immune dysregulation is also the cause of pancytopenia in patients with hemophagocytic lymphohistiocytosis (HLH). This life-threatening syndrome is characterized by impaired pathogen elimination, hyperinflammation, and hystiocytic and lymphoid tissue infiltration. It can occur as familial disease (FHL with *PRF1*, *UNC13D*, *STX11*, or *STXBP2* mutations), in syndromes characterized by additional albinism (i.e., Griscelli syndrome type II, Hermansky-Pudlak syndrome type II, and Chediak-Higashi syndrome) and secondary to infection, rheumatic, or neoplastic disorders. Secondary HLH is also known as macrophage activation syndrome (MAS). Deregulation of T and NK cell cytotoxicity and/or lysosomal trafficking are underlying mechanisms of HLH (43). Pancytopenia is not mediated by autoantibodies but instead by macrophage hyperactivation, resulting in hemophagocytosis and cytokine-mediated marrow suppression. Accordingly, hemophagocytosis in BM is one of the diagnostic criteria, next to hypertriglyceridemia, hypofibrinogenemia, and increased levels of ferritin and soluble IL2 receptor. Degranulation and cytotoxicity assays as well as genetic analysis confirm the diagnosis (44).

## Extrinsic Conditions Associated with Impaired Hematopoiesis

Certain extrinsic, environmental conditions can interfere significantly with blood formation, either pre- or postnatally. The most frequent causes of impaired hematopoiesis are infections. Congenital TORCH infections (i.e., toxoplasmosis, rubella, cytomegalovirus, herpes simplex, and others) often result in decreased maturation of megakaryocytes and platelet formation, in combination with increased immune-mediated platelet destruction (45, 46). Parvovirus B19 infections lead to apoptosis and cell cycle arrest in infected fetal erythroblasts, thereby resulting in fetal anemia and hydrops (47). Also postnatally, parvovirus B19 can transiently affect erythroid progenitors. While healthy children are only mildly affected, children with hemolytic anemia and immunocompromised patients might develop aplastic crisis and persistent anemia, respectively (48). Many other viral infections are associated with transient hematopoietic depression of one or more lineages. Important infections to be considered in the differential diagnosis of peripheral cytopenias are hepatitis C, HIV (in children nowadays mostly due to vertical transmission), and *Helicobacter pylori*, all causing thrombocytopenia and some of them anemia. Visceral leishmaniasis, in contrast, results in an HLH-like disorder with massive phagocytosis of blood components (49, 50).

Other causes of suppressed hematopoiesis are nutritional deficiencies observed in anorexia nervosa or B12, folate, and iron deficiency. While mild deficiencies frequently cause anemia, severe lack of essential food components can result in complete BM failure and immunodeficiency (51–53).

Finally, hematopoiesis can be restricted by pathological microenvironmental conditions. In Gaucher disease, BM dysfunction is caused by accumulation of glucocerebrosides in macrophages and results in (pan)cytopenia (54). By contrast, osteopetrosis is caused by reduced bone resorption leading to bone sclerosis and, among other symptoms, restriction of the medullar cavity. This results in BM failure and compensatory extramedullary hematopoiesis. Different disease subtypes have been reported, with infantile malignant osteopetrosis being the most severe form (55).

## Inherited Intrinsic Defects of Hematopoiesis

Various inborn genetic defects result in hematopoietic failure, and most of them are not restricted to hematopoiesis but also affect different organ systems. Traditionally, Fanconi anemia (FA), dyskeratosis congenita (DC), congenital amegakaryocytic thrombocytopenia (CAMT), thrombocytopenia with absent radii (TAR), Diamond Blackfan anemia (DBA), Shwachman Diamond syndrome (SDS), and severe congenital neutropenia (SCN) have been subsumed as inherited BM failure syndromes (IBMFS). BM failure can occur at different ages and has highly variable presentations. Disease kinetics and degree of severity depend on the underlying gene mutation and presumably on concomitant polymorphisms and environmental influences, such as life style, infections, and exposition to toxins (56). Underlying pathogenetic mechanisms are heterogeneous and comprise metabolic dysfunction, inhibition of differentiation, ribosomal dysfunction, DNA repair deficiency, and telomere maintenance.

In DBA, SDS, SCN, CAMT, and TAR, primarily one hematopoietic lineage is affected. These syndromes, their underlying pathogenesis, and phenotype, as well as characteristic BM findings are summarized in Table 1. However, it has to be kept in mind that patients with CAMT or SDS frequently show a progress from isolated thrombocytopenia or neutropenia, respectively, to complete BM failure (31). In DBA, erythropoiesis is primarily affected but full BM failure also occurs at low frequency (26, 56). Ribosomal dysfunction has been shown to induce excessive TP53 activation leading to apoptosis and being most harmful to highly proliferating erythroid progenitor cells (24, 26).

In the following, we will focus on syndromes affecting more than one hematopoietic lineage and frequently resulting in severe BM failure and pancytopenia. One of the most severe forms of hematopoietic failure is reticular dysgenesis, a rare, autosomal-recessive syndrome. Erythro- and megakaryopoiesis have been described to be unaffected in some patients, but newborns present with complete absence of neutrophils and early onset SCID. Due to the lack of both innate and adaptive immune functions, reticular dysgenesis is rapidly fatal unless hematopoietic stem cell transplantation (HSCT) is performed. Mutations in the *AK2* gene have been identified to be causative for some cases, but most remain unsolved (57, 58).

The most frequent syndromes characterized by severe BM failure are FA and DC. In FA, mutations are found in 15 different genes (*FANCA*, *FANCC*, *FANCG*, *FANCD2*, and others), all involved in DNA damage repair, particularly in resolution of DNA interstrand cross-links during replication. Patients may present with congenital abnormalities, such as short stature, microphthalmia, thumb and radius deformities, café-au-lait spots and heart, renal and genitourinary malformations. Hematopoietic failure often emerges in childhood or adolescence, and most patients are only diagnosed with FA at the onset of pancytopenia (59). When treated with mitomycin C, FA cells accumulate chromosomal breaks and undergo G2 arrest, which is used as a diagnostic test (60). DC is a disease caused by mutations in genes affecting telomere elongation and maintenance (*DKC1*, *TERC*, *TERT, TINF2*, and others). Although the disease was first characterized by its mucocutaneous symptoms (i.e., skin pigmentation, nail dystrophy, and mucosal leukoplakia), it is now known that the development of BM failure may preceed the mucocutaneous manifestations. Age of onset and disease phenotype are highly variable and at least partially depend on characteristic mutations (61). By contrast, the diagnostic work-up invariably reveals very short telomeres at time of presentation. Unresolved DNA damage and critically short telomeres induce DNA damage checkpoint activation in FA and DC cells, respectively, with TP53 being a critical mediator (62). As a consequence, cells stop proliferation, and senescence or apoptosis are induced. Together, these pathways confer protection from malignant transformation but at the same time they contribute to BM failure and pancytopenia. As a consequence, FA and DC cells have a high selective pressure to inactivate the DNA damage checkpoint, as reflected by their high propensity to develop secondary MDS and AML (Tables 2 and 3) (63). The highest risk is observed in FA patients where accumulation of DNA damage and unresolved chromosomal aberrations rapidly lead to malignant transformation (64).

**Table 2:** **Susceptibility to malignancies in inherited bone marrow failure syndromes (7, 65–71)**.

Inherited bone marrow failure syndrome	Reported malignancies
**Fanconi anemia (FA)**	Myelodysplastic syndromes, acute myeloid and lymphatic leukemia, head and neck squamous cell carcinoma, vulva, esophageal, breast and skin carcinoma, brain tumors, basal cell carcinoma
**Dyskeratosis congenita (DC)**	Myelodysplastic syndromes, acute myeloid leukemia, non-Hodgkin lymphoma, head and neck squamous cell carcinoma, cervix carcinoma, basal cell carcinoma
**Diamond-Blackfan anemia (DBA)**	Myelodysplastic syndromes, acute myeloid leukemia, colon and lung carcinoma, basal cell carcinoma, osteogenic sarcoma, female genital cancers
**Shwachman-Diamond syndrome (SDS)**	Myelodysplastic syndromes, acute myeloid leukemia, pancreatic ductal adenocarcinoma (*n* = 1)
**Cartilage-hair hypoplasia (CHH)**	Non-Hodgkin lymphoma, Hodgkin lymphoma, chronic lymphatic leukemia, squamous cell carcinoma, basal cell carcinoma
**Thrombocytopenia absent radius syndrome (TAR)**	Acute myeloid leukemia
**Congenital amegakaryocytic thrombocytopenia (CAMT)**	Acute myeloid leukemia, acute lymphoblastic leukemia
**Severe congenital neutropenia (SCN)**	Myelodysplastic syndromes, acute myeloid leukemia
**Familial platelet disorder (FPD)**	Myelodysplastic syndromes, acute myeloid leukemia, myeloproliferative neoplasms, acute T lymphoblastic leukemia

**Table 3:** **The different pathophysiological mechanisms and their relative contributions to inherited bone marrow failure syndromes, severe aplastic anemia, and myelodysplastic syndromes**.

Pathophysiological mechanism	Inherited bone marrow failure syndromes (IBMFS)	Myelodysplastic syndromes (MDS)	Severe aplastic anemia (SAA)
**Inherited cell-intrinsic defects**	– **Causative for all forms of ÍBMFS** – TP53 activation in FA, DC, and DBA cells results in cell cycle inhibition, senescence, and apoptosis meant to protect from malignant transformation but at the same time contributing to BM failure and pancytopenia (72, 73)	– Familial cases are caused by *RUNX1* or *GATA2* mutations leading to deregulation of the stem and progenitor cell function (74, 75) – No inherited mutations are known for sporadic cases	– No evidence
**Acquisition of driver mutations**	– Secondary evolution to MDS and/or AML (65) – Transformation driven by cumulative injury of proliferating cell (e.g., accumulation of DNA damage or chromosomal instability) (64) – Compensatory proliferation and selective pressure in pancytopenic patients contribute to transformation (64)	– **Driving force for MDS development and evolution to AML** – Typical driver mutations conferring clonal advantages affect the *ASXL1*, *EXH2*, *IDH1*, *IDH2*, *KRAS*, *NRAS*, and *TET2* genes (76) – Such mutations can result in clonal hematopoiesis even before overt MDS and AML occurs (77) – Also, familial MDS forms require second hits (e.g., monosomy 7 in patients with *GATA2* mutation or loss of heterozygosity in patients with *RUNX1* mutations) (78, 79)	– Secondary evolution to MDS – Critical drivers of clonal evolution are compensatory proliferation in the hypocellular marrow and immune escape (64) – Characteristic clonal findings: PNH clones with *PIGA* mutations, deletion of (antigenic) HLA alleles by loss of the chromosomal arm 6p or typical MDS mutations (*ASXL1*, *DNMT3A*, *TET2*, and others) (80–82)
**Autoimmunity**	– No contribution reported (83)	– Clonal T cells were found in MDS patients. Conflicting results indicate that they are either derived from the MDS clone or have been induced by antigenic mutations in MDS cells (84, 85) – Approximately 10% of MDS patients have autoimmune-inflammatory manifestations, but the pathophysiological relationship between MDS and autoimmunity remains unclear (86) – Some patients show hematological recovery upon immunosuppressive therapy (87)	– **Primary event for SAA** – Mediated mainly by CD8+ cytotoxic T and Th1 cells that are recruited to the BM (88, 89) – Association with certain HLA alleles (90, 91) – Autoantibodies have been identified but their significance remains unclear (92, 93) – Patients show a good response to immunosuppressive therapy (94, 95)
**Inflammatory signaling**	– Inflammation and infectious diseases are thought to accelerate BM failure. Repeated interferon stimulation induces BM failure in a Fanconi mouse model (96) – Cytokines induce proliferation and subsequent exhaustion of stem cells. Cycling stem cells get more susceptible toward apoptosis (96, 97)	– Overproduction of cytokines (i.e., TNFα and IFNγ) by the stem cell niche contributes to apoptosis of MDS cells (98)	– Th1-shifted cytokine secretion with (i.e., IFNγ, TNFα, and IL-2) contributes to disease pathogenesis by suppressing hematopoiesis (99–101)
**Deregulation of the stem cell niche**	– There is evidence that the function of the stem cell niche is compromised because of the underlying genetic mutation (102, 103) – However, allogeneic stem cell transplantation can correct all hematological symptoms indicating a minor contribution to disease by the microenvironment	– Microenvironmental deregulation contributes to pathogenesis (98, 104) – In animal models, niche alterations can be sufficient to induce MDS (i.e., by Dicer mutations) (105) – Clonal hematopoiesis remodels the niche: in MDS xenograft models, healthy mesenchymal stromal cells cotransplanted with MDS cells adopt molecular features observed in mesenchymal stromal cells derived from MDS patients (106)	– There is evidence that the stem cell niche might contribute to T cell activation; the results however are conflicting (88)

*AML, acute myeloid leukemia; BM, bone marrow; DBA, Diamond Blackfan anemia; DC, dyskeratosis congenita; FA, Fanconi anemia; PNH, paroxysmal nocturnal hemoglobinuria; SDS, Shwachman Diamond syndrome*.

Other genetic diseases associated with pancytopenia are Seckel and Pearson syndromes. Seckel syndrome represents a DNA damage repair deficiency characterized by BM failure, dwarfism, microcephaly, mental retardation, and skeletal malformations. Among others, *ATR* mutations have been identified to be responsible for this rare syndrome (107). Pearson syndrome is caused by loss of mitochondrial DNA and thus one of the so called mitochondriopathies. Next to failure to thrive, exocrine pancreatic dysfunction, and susceptibility to metabolic imbalance, patients frequently suffer from anemia, thrombocytopenia, and/or neutropenia. BM analysis typically shows sideroblastic anemia and vacuolization of hematopoietic precursor cells (108).

It is hardly surprising that DNA damage deficiencies, telomeropathies, ribosomopathies, or mitochondriopathies do not only disturb hematopoiesis but also affect developmental programs and adult tissues. Many of the syndromes described here are additionally characterized by an increased susceptibility to malignancies, including hematological malignancies and solid tumors (Table 2) (65–71). It is obvious that both, the multisystemic nature of disease and the tumor susceptibility, have to be taken into account during patient care and therapy planning. It is therefore of great importance to recognize the underlying cause of BM failure even in patients with mild or no extrahematological symptoms (56, 109). Pitfalls and diagnostic approaches especially in disorders with hypocellular BM are discussed below.

## Acquired Disorders of Hematopoiesis

### Myelodysplastic syndrome

Myelodysplastic syndrome (MDS) is a clonal disorder originating from a hematopoietic stem/progenitor cell that supposedly has acquired driver mutations. Due to deregulation of differentiation and increased susceptibility to apoptosis, MDS is characterized by ineffective hematopoiesis in one or more lineages. BM analysis shows characteristic dysplastic cells, such as micromegakaryocytes, binucleated erythroid precursors, and hypo- or hypersegmented neutrophils. MDS has a high propensity to further progress to more advanced disease, including MDS-related acute myeloid leukemia (MDR-AML). This evolution is caused by additional subclonal driver mutations that have been acquired in a stepwise manner and result in further increase in proliferation and impairment of differentiation (110).

In general, MDS is a disease of old age, with an incidence of 50 cases per 100,000 persons aged over 70 years. As shown recently, even healthy, aged individuals frequently have clonal expansion of hematopoietic cells harboring driver mutations (i.e., *DNMT3A*, *JAK2*, *TET2*, and others), although they do not (yet) suffer from overt MDS or leukemia (77). Risk of MDS is further increased in people exposed to chemo- or radiotherapy earlier in life with a life-time risk of 2–10% after treatment with alkylating agents, topoisomerase II inhibitors, or ionizing irradiation (111).

By contrast, childhood MDS is rare, and differs from adult MDS in several aspects. For this reason, the WHO 2008 classification incorporated a classification specific for childhood MDS allowing unambiguous classification of most patients (112). Depending on the number of blasts in peripheral blood (PB) and BM, childhood MDS is classified as refractory cytopenia of childhood (RCC; PB blasts <2%, BM blasts <5%), refractory anemia with excess blasts (RAEB; PB blasts 2–10%, and/or BM blasts 5–19%), RAEB in transformation (RAEB-T; PB and/or BM blasts 20–29%), and MDR-AML (>30% BM blasts) (113). The most common subtype is RCC (50%). While adult MDS patients usually present with isolated anemia (“refractory anemia”), affected children frequently present with thrombocytopenia (<150,000/μl; 75%), neutropenia (<1,000/μl; 50%), and/or anemia (Hb <10 g/dl; 50%). HbF and MCV are frequently elevated (114). In >80% of all RCC cases, BM analysis reveals a marked decrease of cellularity. The remaining 20% of patients with RCC have a normo- or hypercellular BM (114).

Common cytogenetic abnormalities of childhood MDS are monosomy 7 (approximately 30%) and trisomy 8. Loss of chromosome 5q, frequently seen in adults, is only rarely found in children. A structurally complex karyotype (≥3 chromosomal aberrations including at least one structural aberration) is rare but invariably associated with poor prognosis (115–117). Chromosomal aberrations do not represent initiating events but indicate disease progression. Along this line, karyotypic evolution is usually accompanied by progression to more advanced MDS forms. The incidence of cytogenetic abnormalities is lowest in RCC with hypocellular BM. Patients with RCC and monosomy 7 are at high risk of progression with a cumulative incidence of 80% at 6 years from diagnosis and a median time to progression of 1.9 years (118). By contrast, RCC patients with a normal karyotype or trisomy 8 may have stable disease for many years. Patients with advanced MDS are at risk for further disease progression and have an indication for early HSCT (114). In infants, monosomy 7 or del(7q) has also been reported to disappear spontaneously, however, only in rare cases (119).

Next to sporadic cases, MDS can arise secondary to inherited or acquired BM disorders or in the context of chemo/radiotherapy. Importantly, all IBMFS described above have a risk to transform into secondary MDS, as described above in more detail for FA and DC (Table 2) (64). Lifetime risk of IBMFS patients is highly variable and ranges from very high in patients with FA (40–50% by the age of 40 years) to very low in DBA patients (65). Patients with other inherited syndromes might develop MDS directly without hypocellular prophase (120). *GATA2* haploinsufficiency is the most frequent reason for such familiar MDS. Patients show variable disease complexes with extrahematological symptoms, such as lymphedema and deafness, and 40% develop MDS during adolescence and early adulthood. Preceding hematological and immunological symptoms are heterogeneous and include monocytopenia, mild neutropenia, and DC, B and/or NK cell deficiency. Depending on the predominant symptoms, the syndrome was named DCML (dendritic cell, monocyte, B and NK lymphoid) deficiency, Emberger syndrome, or MonoMAC (121). Similarly, patients with heterozygous germline *RUNX1* mutation have a high propensity to develop MDS (20–50%). Since precedent hematological symptoms are limited to qualitative and quantitative platelet defects, the syndrome is called familial platelet disorder (FPD) (33). It is not yet fully understood, why patients with *RUNX1* and *GATA2* mutations are at such high risk for MDS (120).

### Severe aplastic anemia

While MDS is an intrinsic disorder of hematopoiesis, severe aplastic anemia (SAA) is an autoimmune process. In contrast to autoimmune cytopenias affecting mature blood cells, immature and multipotent cells are targeted in SAA resulting in the severe phenotype characterized by pancytopenia and BM aplasia (88, 94). Evidence for the immune pathophysiology of the disease was first derived from clinical observations such as the response to immunosuppressive therapy (IST) or successful autologous recovery after failed allogeneic HSCT (122, 123). Since then, much effort has been invested in characterization of the immune phenotype, and certain HLA alleles have been associated with an increased risk (90, 91). Autoantibodies against different antigens (e.g., antimoesin or kinectin) have been detected in SAA patients but their significance remains unknown (92, 93). Detailed analysis of the T cell compartment revealed a recruitment of activated T cells to the BM (124) and oligoclonality in some cases (125–128). CD8+ cells isolated from SAA patients were able to suppress colony formation *in vitro* (129). Th1-shifted cytokine secretion with elevated IFNγ, TNFα, and IL-2 levels is thought to contribute to disease pathogenesis by suppressing hematopoiesis (89, 99–101).

Although SAA is primarily immune-mediated, acquisition of somatic mutations and clonal hematopoiesis seem to be relevant phenomena. Clonal evolution is thought to be driven mainly by two mechanisms: (i) proliferative pressure to compensate for hematopoietic failure, and (ii) immune escape.

Best described is the presence of a so-called paroxysmal nocturnal hemoglobinuria (PNH) clone in patients with SAA. In PNH, the clonal cell population harbors a mutation in the *PIGA* gene and thus lacks cell surface proteins linked to a glycosylphosphatidylinositol anchor. In clinically manifest PNH, this leads to complement-mediated hemolysis and the risk of thrombosis (80). It is possible that *PIGA* mutations occur to escape autoimmunity directed against glycosylphosphatidylinositol-bound antigens. Alternatively, the clone might exist *a priori* and be positively selected when other hematopoietic cells are hit by autoreactive T cells in SAA. Also, copy number-neutral loss of heterozygosity of chromosome 6p is observed disproportionately frequent in SAA patients (81). This is thought to be a mechanism of immune-escape by deletion of antigenic HLA alleles. Finally, SAA patients have been shown to carry somatic mutations similar to those mutated in MDS (i.e., *ASXL1*, *DNMT3A*, *TET2*, and others), indicating transformation into secondary MDS (82, 130).

### B-cell precursor acute lymphoblastic leukemia

About 2% of patients with B-cell precursor acute lymphoblastic leukemia (ALL) present with pancytopenia and hypocellular BM. Usually, this prophase is transient and goes into spontaneous remission before ALL emerges with a latency of up to 9 months. Hematopoietic cells in the aplastic phase have been shown to carry the same gene mutations as the leukemic blasts (131, 132).

## Inherited Bone Marrow Failure, MDS, and SAA: The Diagnostic Challenge of a Hypocellular Marrow

One of the major challenges in pediatric hematology is the correct diagnosis of patients with pancytopenia and hypocellular BM. Once infections and other more frequent conditions resulting in a transient suppression of hematopoieisis have been excluded, the main differential diagnosis includes SAA, hypoplastic MDS, and IBMFS, such as FA and DC. Careful diagnostic work-up and accurate distinction of these entities are mandatory because they require different strategies for therapy, surveillance, and counseling. In particular, IBMFS have to be excluded since they often present as multisystem and tumor-susceptibility syndromes with limited tolerance to various therapies (56, 64, 109). The diagnostic challenge is not only due to similar clinical presentation but also caused by a relevant overlap in the underlying pathophysiology. Table 3 shows different pathophysiological mechanisms contributing to IBMFS, MDS-RCC, and SAA to variable degrees. Further problems for the diagnostic work-up are scarce cell numbers isolated from hypocellular BM and lack of discriminating functional tests.

Knowing these hurdles, the following diagnostic criteria and workflow should be followed:

(i) BM analysis: next to aspiration cytology, histological analysis of a trephine biopsy should be performed. RCC and SAA can be reliably differentiated in trephine biopsies (114, 133) and the respective criteria, as defined in the WHO classification 2008, are depicted in Table 4 (82, 113). Additionally, cytogenetic analysis with metaphase cytogenetics and FISH should be performed. Chromosomal aberrations indicate the presence of MDS. However, >60% of all RCC cases have a normal karyotype (114). Next generation sequencing will be helpful to detect clonal driver mutations indicative for MDS-RCC, but this method is still expensive and reserved for research questions.
(ii) Exclusion of IBMFS: IBMFS and RCC frequently have overlapping morphological features. Thus, careful past medical and family history, as well as physical examination, is required to exclude presence of a multisystem disease. FA and DC should be excluded by mitomycin C stimulation and telomere measurements, respectively. Genetic testing can confirm the suspected diagnosis, but gene mutations cannot be detected in all cases. In case of the diagnosis of a defined IBMFS or a presentation that is highly suspicious for an inherited disorder, possible related stem cell donors should be carefully evaluated for signs and symptoms of the respective disease. Patients with strong evidence of multisystem disease but without genetic diagnosis might benefit from functional tests such as chromosomal breakage, cell cycle arrest, ribosomal profiling, or others. However, such tests might be technically challenging and difficult to interpret and thus are not part of standard diagnostic work-up.

**Table 4:** **Morphological criteria for severe aplastic anemia and myelodysplastic syndromes, type refractory cytopenia of childhood (EWOG-MDS 2008) (13, 82, 113)**.

	Myelodysplastic syndrome, type refractory cytopenia of childhood (MDS-RCC)	Severe aplastic anemia (SAA)
**Erythropoiesis**	Patchy distribution, increased numbers of proerythroblasts (left shift), increased numbers of mitoses	Lacking or single small focus with <10 cells, full maturation
**Granulopoiesis**	Marked decrease, left shift	Lacking or marked decrease, very few small foci with maturation
**Megakaryopoiesis**	Marked decrease or absence Micromegakaryocytes (to be detected by immunohistochemistry)	Lacking or very few, no micromegakaryocytes or other dysplastic megakaryocytes
**Lymphocytes**	Lymphocytes and plasma cells might be increased	May be increased focally or dispersed
**CD34***+*** cells**	No increase	No increase
**Others**	Mast cells might be increased Adipocytic bone marrow No increase in reticulin fibers (difference to adult MDS patients)	After initation of immunosuppressive therapy: similar to MDS-RCC

Since disorders of hypocellular BM are rare, diagnostic work-up should be performed by experienced hematologists and hematopathologists, and patients should be treated after consultation with reference centers. This ensures best care for affected patients and their families and prevents underdiagnosis of RCC and telomeropathies in SAA cohorts (109, 134–136).

It is also important to repeatedly reevaluate patients with IBMFS and SAA, since both disorders are at risk to progress into secondary MDS. Secondary MDS can be diagnosed by an increase in blast cells or BM cellularity, despite persistent pancytopenia, and by acquisition of chromosomal aberrations.

## Therapeutic Approaches and Limitations

Treatment of (pan)cytopenia is based on a few symptomatic or curative therapeutic options. Symptomatic treatment includes the transfusion of blood products, such as red blood cells and platelets, and prevention or treatment of infections with antimicrobial drugs, and if indicated immunoglobulin substitutions. Granulocyte or T cell transfusions are technically feasible but restricted to very few life-threatening situations. Hematopoietic growth factors may be used to stimulate hematopoiesis and overcome reduced blood cell production. G-CSF is the therapy of choice for patients with SCN and has changed the fate of majority of children with this serious condition (19, 137). Today, allogeneic HSCT is limited to patients who do not respond to G-CSF or develop MDS (138). Although the situation is less clear, selected patients with SDS might benefit from G-CSF in case of severe neutropenia (66). The thrombopoietin analog eltrombopag has been successfully used in chronic ITP (139) and surprisingly induced multilineage responses in an early study in patients with refractory SAA (140). Other, less specific stimulants of hematopoiesis are the steroid prednisolone or the androgens, danazol and oxymetholone, used for DBA or DC, FA, and SAA patients, respectively (26, 141–144).

Some of the disorders described in this review require immune-modulatory or suppressive therapies. These include high dose-immunoglobulins, anti-thymocyte globulin (ATG), steroids, cyclosporine, mycophenolic acid, and others. Detailed treatment schedules for ITP, AIHA, ALPS, SLE, HLH, and others are beyond the scope of this review and described elsewhere.

Immunosuppressive therapy traditionally has been the therapy of choice for patients with SAA with no matched related donor (94, 145). The best established regimen consists of ATG in combination with cyclosporine A (95). In 2011, a randomized trial demonstrated that the source of ATG (horse vs rabbit) is relevant and the treatment with ATG derived from horses is more effective compared to products derived from rabbits (146). These results have subsequently been confirmed in multiple observational studies (147, 148). Using the combination of horse ATG and cyclosporine, the response rate is approximately 60–70% with a risk of relapse or clonal evolution of 10–30 or 10–20%, respectively (145). Attempts to improve efficacy were based on additional treatment with sirolimus, mycophenoalte mofetil, or danazol, and did not result in significant improvements. Also, the addition of G-CSF did not result in better response rates or improved survival, and therefore is not generally recommended. Based on the observation that autoimmunity contributes to the pathophysiology of MDS (Table 3) and that adult patients with hypoplastic MDS have successfully been treated with IST (149), selected pediatric patients with hypocellular RCC have successfully been treated with IST (87, 150, 151).

Hematopoietic stem cell transplantation is a curative treatment for patients with SAA and MDS, and the hematological manifestation of IBMFS. Patients with SAA traditionally were transplanted following a conditioning regimen consisting of cyclophosphamide/ATG in the presence of a matched sibling donor with excellent results (152). Based on less favorable results, HSCT from an unrelated donor was only performed in patients with non-response to IST, relapse, or clonal evolution. Lately, the results of HSCT from unrelated donors have improved considerably and upfront primary HSCT from a well-matched unrelated donor might be considered in selected cases if the donor is available in an acceptable timeframe (153–155).

In RCC, the indication for HSCT is based on the severity of pancytopenia (ANC <1000/μl and/or transfusion dependency) and the presence of cytogenetic aberrations (monosomy 7, 7q-, complex karyotype). Patients with hypocellular RCC with normal karyotype are successfully treated with a reduced intensity regimen, whereas patients with cytogenetic aberrations or a normo-/hypercellular marrow require a more intensive regimen. Patients with advanced MDS should be transplanted as soon as possible following an intensive conditioning regimen (118, 156, 157).

In IBMFS, allogeneic HSCT is able to treat the hematological, and immunological manifestations of the disease. However, extrahematological manifestations, such as pulmonary fibrosis in DC, are not only not treated by this therapeutic approach but may also be an insurmountable obstacle for HSCT. In addition, the majority IBMFS are multisystem disorders with a complex pathophysiology that result in an increased sensitivity to chemotherapeutic drugs requiring a specific adaption of conditioning regimens (158). The careful evaluation of possible related stem cell donors is of special importance in familial disease.

Finally, it has to be mentioned that HSCT is also the only curative treatment for all forms of primary immunodeficiencies and familial HLH. Experimental approaches such as targeted gene correction are under evaluation with regard to efficacy and safety, but not yet suitable for broad application.

In summary, it is still a great challenge to find the correct diagnosis for patients with (pan)cytopenia, especially in the presence of a hypocellular BM. Better definition and differentiation between the individual inherited and acquired disorders are required to ensure adequate medical care, surveillance, and genetic counseling for affected children and their families. To this end, the underlying pathophysiological mechanisms have to be investigated in more detail. Furthermore, functional tests and biomarkers with higher specificity for individual diseases have to be developed. Finally, we recommend that all affected children should be registered in registries and/or clinical trials and treated according to general guidelines.

## Author Contributions

ME and BS wrote the manuscript.

## Conflict of Interest Statement

The authors declare that the research was conducted in the absence of any commercial or financial relationships that could be construed as a potential conflict of interest.
